# An entropy-based automated approach to prostate biopsy ROI segmentation

**DOI:** 10.1186/1746-1596-8-S1-S24

**Published:** 2013-09-30

**Authors:** Gloria Bueno, Maria-Milagro Fernández-Carrobles, Oscar Déniz, Jesús Salido, Noelia Vállez, Marcial García-Rojo

**Affiliations:** 1 VISILAB, E.T.S.Ingenieros Industriales, Universidad de Castilla-La Mancha, Spain; 2 Dpto. Anatomía Patológica, Hospital General Universitario de Ciudad Real, Spain

## Background

Despite significant improvements in computer vision and image processing techniques, there are few software tools that are able to analyze prostate biopsy images in a fully automated way in order to find ROIs in those images. In order to develop a useful system, user interaction should be minimized, and the system should also be capable of dealing with images acquired at least at 10x magnification, since images of lower resolution do not provide enough information for cancer diagnosis.

The segmentation of the ROI mentioned above is a complex task that includes several challenges. The suppression of user interaction means that the system should be robust enough to deal with image irregularities by itself. Such irregularities may include stain intensity variations, tissue cuts, and even dust over the slide when it is digitized. The physical size and memory requirements of the images also limit the processing algorithms that may be used, since we want our system to be used in personal computers (i.e. not clusters).

In recent years, there have been several studies that focus on Hematoxilin and Eosin (HE) prostate biopsy image processing and histological image analysis. Most of them are focused on the segmentation of only one ROI, usually the nucleus and glands, as well as the extraction of descriptors for classification purposes. A thorough review of the research related to classification may be found in Bueno *et al.*[1]. Statistical information techniques, region growing algorithms [2], fuzzy c-means [3] active contour models, including level set methods [4,5], filtering and morphological analysis [2,5,6] have been also used for ROI detection. The main problem with these methods is that they are not designed to process large amounts of data, which is the case when working with whole digital slides in pathology. Besides, many of these methods yield limited results because they focus mainly on a single structure or type of tissue.

None of the previous techniques use complete mosaics or WSI but rather fragments or magnifications lower than 5x, with the exception of Doyle *et al. *[7] and Vidal *et al. *[5]. Doyle *et al.* used 40x images ranging between 1-2 GB and Vidal *et al.* worked with images acquired at low magnification (5x, 10x) and up to 1.19 GB in size. One of the problems with level set methods is that they are not suitable for parallel processing. Moreover, level set methods have not been used in a general way, only applied to one type of histological images HE biopsies). In a recent work by the authors [1], a general solution is described for parallelizing in an efficient way a set of heterogeneous low and high-level image processing algorithms to be applied to high resolution histopathological WSI. The imaging tools implemented in [1] are general for all types of histological images with different stain, acquired from different anatomical parts and digitized at different magnifications. These tools deal with contrast analysis, ROI detection and classification applied to high resolution images that range from 300 MB to 30 GB.

The aim of this system that will be described herein is the segmentation of ROIs from these images in a way that mimics the method used by doctors, that is, identifying at low magnification the regions with high concentration of cells or where the architectural distribution between lumen and cells is relevant. In this way this work differs from those previously mentioned. The method may be applied to different histological images and are suitable for parallel processing, which differs also from the method presented by Vidal *et al. *[5].

## Material and methods

A dataset of 200 biopsies stained with HE and provided by the Department of Pathology, Hospital General Universitario de Ciudad Real (HGUCR) was used. The images were digitized using an ALIAS II microscope, from LifeSpan Biosciences Inc. This system acquires tiles with a size of 2000 x 2000 pixels and 24 bits per pixel (RGB). Each tile requires 11.4 MB. The ALIAS microscope is equipped with five different objectives, whose magnifications are 2.5x, 5x, 10x, 20x, and 40x. We have focused on samples digitized with 10x magnification, although our work could be easily adapted to be magnification-independent. The images at 10x magnification have memory requirements ranging from 8.83 MB (1899 x 1626 pixels) to 220 MB (9755 x 7884 pixels).

The pathologists at HGUCR have also specified the most relevant features that should be considered when analyzing prostate biopsies at these magnifications. HE stained prostate biopsies have three types of well-differentiated structures of interest: lumen, cytoplasm, and cells. For pathological purposes, the most important structures are cells [1]. Their morphology, distribution between them, and relationship with lumen and cytoplasm are the most relevant features that pathologists consider to elaborate a diagnosis. Groups of cells are especially important, and they are what we consider a true ROI. These groups may appear either surrounding a lumen area, or packed very closely. Typically, in both cases some cytoplasm will appear between the cells. Thus, ROI are complex areas, where the three types of structures of interest appear in an unpredictable fashion. Although lumen and cell areas can be individually segmented without much effort, the segmentation of ROIs where the structures are grouped requires advanced techniques. It is desirable that all the three types of structures share a common feature (or a manageable set of them), so they all can be separated from the rest of the image using that feature.

If an RGB image of a prostate biopsy is converted to the YIQ colour model, and then the I channel is extracted from the image and equalized, the result is an image where the regions of interest are clearly highlighted respect to the rest of the image. It is important to apply the equalization only to the region where the tissue lies, since its results vary if it is applied to a region where there is no tissue present.

Once the I channel is properly equalized, regions of interest clearly appear darker than other regions, so a binarization could be used to separate ROI from the rest of the tissue. This binarization sets as foreground all the dark regions in the image (without the non-tissue region), and sets everything else as background. However, this basic technique alone does not produce good results on most images. Since images tend to vary greatly in the edges of the three types of the structures of interest, especially in the outer border of the cells, entropy turns out to be a great feature to determine where the ROIs are located. It has been observed that entropy calculations produce better results when applied to the green channel of the RGB image, because it is the one that features higher contrast between the structures of interest.

Given a pixel in an image, *P_i_* = (*x_i_*,*y_i_*), a circular neighborhood of radius *R* around it is composed by all the pixels *P_j_* = (*x_j_*,*y_j_*) with an Euclidean distance to *P_i_* is lower than *R*. For each pixel *P_i_* in the green channel, a circular neighborhood around it is defined. Then, the histogram of the neighborhood is calculated as well as its entropy. Suppose that *f_i_* is the relative frequency of pixels with intensity value *i*, then the entropy at *P_i_* is calculated as: .

The results of entropy calculation depend on the radius of the neighborhood considered. Obviously, the computational footprint of entropy calculation increases with the size of the neighborhood. The chosen radius for entropy calculation is 27. The result of entropy calculation is also equalized. It should be remarked that entropy calculation, as well as equalization, is only applied to the tissue in the image, and therefore the background does not affect these calculations.

**Figure 1:**
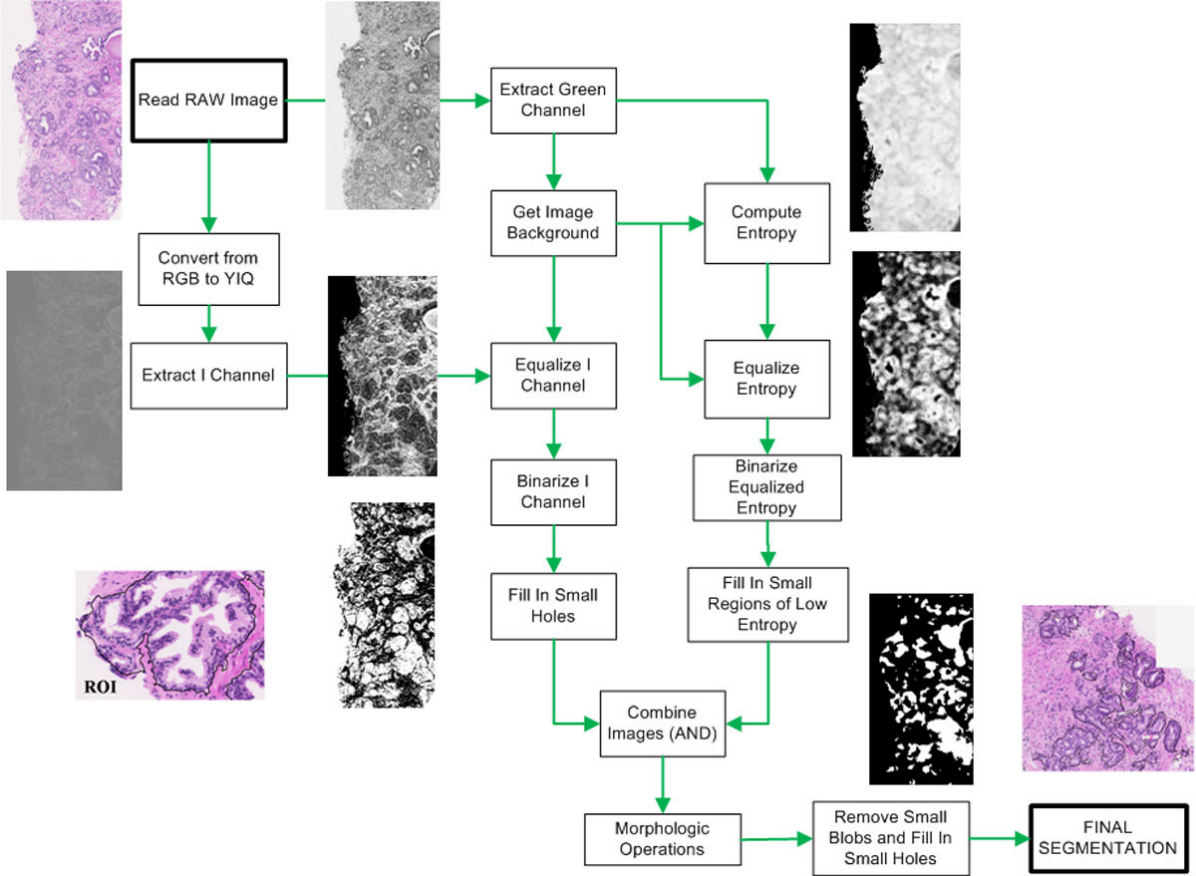
Flowchart of the segmentation process.

The entropy image may be binarized in order to separate regions of high entropy from the rest of the image. Since big lumen areas present low entropy, connected component analysis is performed to fill in the holes that are smaller than a predetermined size. Then, it is combined with the binarized I channel using the logic operator AND. Finally, morphologic operators are used to remove noise and smooth the results. These morphologic operations involve a first dilation with a circular kernel with radius 3, followed by an erosion (circular kernel, radius 5) and a final dilation (circular kernel, radius 2). Figure 1 illustrates the full segmentation process.

## Results and discussion

**Figure 2:**
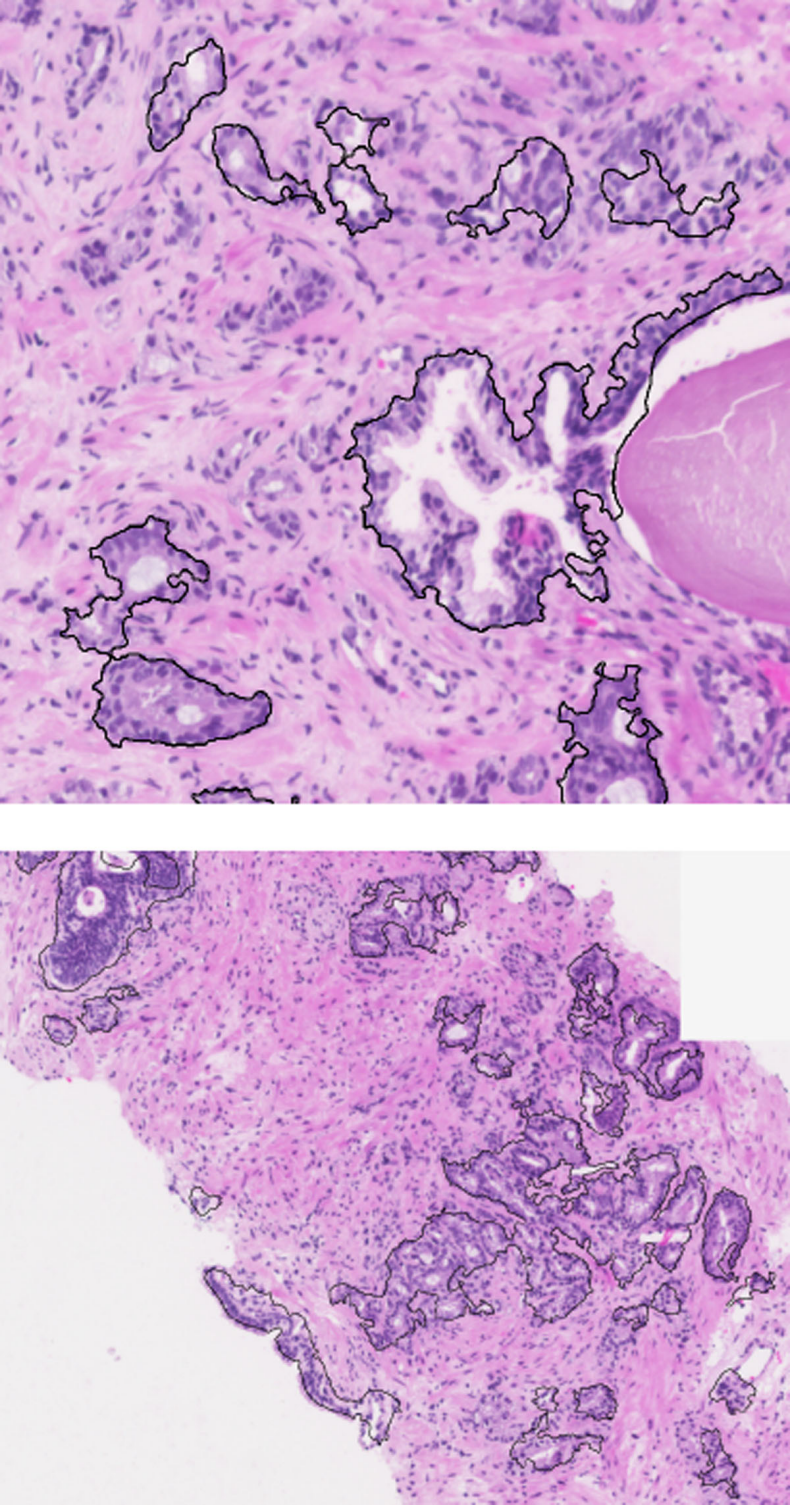
**Examples of segmented region of interests.** Results of the segmentation algorithm applied to prostate biopsy samples digitalized at 10x.

Some selected fragments that exemplify the algorithm are shown in Figure 2. Although the images used to test the algorithm were large, computational times were not deemed excessive. The algorithm takes from 9 seconds to 5 minutes for 9MB (1899x1626 pixels) and 220MB (9755x7884 pixels) images respectively. The test machine was equipped with an Intel Pentium 4 640 (3.2 GHz) and 2GB RAM (DDR2-533MHz).

A quantitative validation based on ROC analysis was carried out with our set of 100 different tissue samples of WSI. The samples were both benign and malign samples of prostate biopsy. The results of the algorithm were compared to the manual selection of ROIs done by pathologists from the HGUCR. Thus, the rates of true positive (TP), true negative (TN), false positive (FP), and false negative (FN) detections were calculated. On average, 15% of detections were FN, 2% were FP, 85% were TP, and 98% were TN. The type I error, that is, the FP for the 100 images, occurs in those samples stained with weak HE dye. The final results show an average sensitivity of 83% with specificity above 99%.

## Conclusions

In this paper, an approach to ROI segmentation in whole slide images of prostate biopsies has been described. The method proposed is based on texture and colour based analysis.

The novelty of the technique lies in the ability to detect complete ROIs, where a ROI is composed by the conjunction of three different structures, that is, lumen, cytoplasm, and cells with a high density of cells and the architectural distribution between lumen and cells. The method is capable of dealing with full biopsies digitized at different magnification. The proposed algorithm is also original because it works on large images acquired with low magnification, thus being different from other algorithms that require higher magnification and have been tested only on small samples. In this way, the method tries to mimic the manual procedure of expert clinicians. Moreover, the method is suitable for parallelization and may be applied to different tissue samples.

The proposed system is also useful because it can be used for different purposes. It could be integrated into a slide visualization environment to highlight the ROIs for the pathologists, either for slide analysis or even with teaching purposes. The system could also be used as a previous step in classification applications, since it could reduce the amount of information to be processed, and probably speed up the whole classification process.

Segmentation accuracy is high for HE stained samples. Furthermore, the algorithm is also reasonably fast. We are currently working to improve the robustness and speed of the algorithm, making it less sensitive to disturbing factors such as different illumination conditions, tissue thickness, and stain amount, as well as parallelizing some parts of the pipeline in order to speed it up. We are also working in developing a set of features of interest that should be segmented and analyzed in order to provide further information to the doctors.

## Competing interests

The authors declare that they have no competing interests.

## Authors' contributions

All authors from VISILAB have developed and tested the algorithm. MG-R from HGUCR has provided the tissue samples and qualitatively validated the results. All authors contributed equally in writing the manuscript.
